# Tea consumption and serum uric acid levels among older adults in three large-scale population-based studies in China

**DOI:** 10.1186/s12877-021-02216-8

**Published:** 2021-04-21

**Authors:** Dan-Dan Chen, Xing-Xuan Dong, Xue-Jiao Yang, Hong-Peng Sun, Gang Liang, Xing Chen, Chen-Wei Pan

**Affiliations:** 1 State Key Laboratory of Radiation Medicine and Protection, School of Radiation Medicine and Protection, Soochow University, Suzhou, 215123 China; 2 Collaborative Innovation Center of Radiological Medicine of Jiangsu Higher Education Institutions, Suzhou, China; 3 School of Public Health, Medical College of Soochow University, 199 Ren Ai Road, Suzhou, 215123 China; 4 Department of Ophthalmology, the Affiliated Hospital of Yunnan University, Kunming, China; 5 Department of Ophthalmology, the Second People’s Hospital of Yunnan Province, Kunming, China; 6 Department of Children Health Care, the Affiliated Suzhou Hospital of Nanjing Medical University, No. 26, Dao Qian Road, Suzhou, 215000 China

**Keywords:** Tea drinking status, Older adults, Serum uric acid, SUA

## Abstract

**Background and aims:**

The association between serum uric acid (SUA) and tea consumption has been studied in previous work, and there were arguments among various population group employed as well as different statistical approaches. The aim of this work is to investigate the tea effect on SUA levels among older adults by comparing three large-scale populations with both cross-sectional and longitudinal analyses.

**Method:**

We examined the relationship between intake and SUA levels among older adults using linear regression. All the studies include the parameters SUA levels, tea intake, age, sex, education level, smoking status, alcohol drinking status, body mass index (BMI), and health history (diabetes, hypertension, and fasting plasma glucose). The cross-sectional analyses were conducted with 4579 older adults in the Weitang Geriatric Diseases Study (WGDS, ≥60 years), 2440 in the China Health and Nutrition Survey (CHNS, ≥60 years) and 1236 in the Chinese Longitudinal Healthy Longevity Survey (CLHLS, ≥62 years); and the longitudinal analyses were performed with 3870 (84.5%) in the WGDS and 420 (34.0%) in the CLHLS. Multivariable linear regression analyses were performed in both cross-sectional and longitudinal studies.

**Results:**

Cross-sectional studies showed that tea consumers tended to have higher SUA levels than non-tea consumers in all the three datasets (*P* < 0.05). However, longitudinal associations of SUA levels with tea consumption had no statistical significance (P>0.05). The results of sex-stratified analyses were consistent with those of the whole datasets.

**Conclusions:**

This work implied that any possible association between tea consumption and SUA levels could be very weak.

## Background

Hyperuricemia (HUA) is a major risk factor for several chronic conditions such as gout [1], cardiovascular diseases (CVDs) [2], diabetes [3], and hypertension [4]. HUA is also associated with increased hospitalization risks and healthcare expenditures [5]. The prevalence of HUA has been increasing throughout the world [6]. It was estimated that approximately 21.4% of American adults suffered from HUA [6], and the prevalence of HUA ranged from 13 to 25.8% in Asians [7–10]. Thus, identifying modifiable risk factors of HUA is crucial for addressing this health public concern.

Tea (*Camellia sinensis*) is a common and popular beverage worldwide, especially in Asia [11, 12]. The association between tea consumption and levels of serum uric acid (SUA) was inconsistent among different studies. For example, some studies supported that tea consumption could reduce SUA levels in both human beings [13] and animals [14, 15]. However, other studies found that tea [16] or its extracts [17] were associated with a higher level of SUA. Non-significant association between tea consumption and SUA levels was also reported in previous research [18]. Thus, the impact of tea consumption on levels of SUA is far from conclusive and needs further clarifications.

Considering that HUA is a common chronic condition in the elderly, who are fond of drinking tea more than the young, understanding the health impact of tea consumption among older adults has important public health implications. Large-scale population-based studies, especially cohort studies, could provide cogent evidence on the relationship between exposures and outcome measures [19]. In the present analysis, we examined both cross-sectional and longitudinal associations between tea consumption and SUA levels in three large-scale datasetsof mainland China (the WGDS, the CHNS and the CLHLS). The findings would further elucidate the role of some tea-related nutrients (e.g., polyphenols) in the etiology of SUA-related conditions (e.g. gout) and help to design appropriate prevention strategy.

## Methods

### Study design and datasets

The WGDS was conducted in Weitang town of Suzhou among all old adults aged 60 years or above [20, 21]. The participants had been living in the town for more than 6 months, not moved from the residing address, and not been found deceased. The baseline survey was conducted in 2014, when 4579 participants completed clinical examinations and provided blood samples [22, 23]. For the 4-year follow-up, 709 participants were excluded who died prior to the follow-up study, could not be contacted, or declined to participate. Therefore, 3870 (84.5%) participants remained during the follow-up examination in 2018. Participants reported their tea-drinking status (Yes/No based on whether drinking tea habitually), frequencies over the past 12 months (≤1time/week, 2–3 times/week, 4–5 times/week and 6–7 times/week), and tea types (green tea, black or oolong tea and other). In the WGDS, a chemistry analyzer (Roche cobas c 501, Switzerland) was used to detect SUA levels (μmol/L). Both cross-sectional and longitudinal analyses were performed in the WGDS.

The CHNS was an international collaborative project between the Carolina Population Center at the University of North Carolina at Chapel Hill and the National Institute for Nutrition and Health at the Chinese Center for Disease Control and Prevention (CCDC) [24]. The survey was conducted in 15 provinces and municipal cities in mainland China. The dataset of the year 2009 was chosen due to SUA collection only in this year. There were 11,929 participants attending in the survey in 2009, but this study included 2440 participants who was considered “eligible” for the age criterion (no less than 60 years old). Participants reported their frequencies of tea consumption in questionnaires. Categories of tea intake frequencies were “daily” (at least one serving per day), “weekly” (less than one serving per day but at least one serving per week), “monthly” (less than one serving per week but at least one serving per month), and less than monthly or none. If a participant reported that he or she drank tea less than monthly or none, he or she was defined as a non-tea consumer. SUA levels (μmol/L) were examined using a chemistry analyzer (Hitachi 7600, Japan). Only the cross-sectional analysis was performed in CHNS due to the lack of longitudinal data.

The CLHLS was a dynamic cohort study conducted by the Center for Healthy Aging and Development Studies of Peking University [25]. The study samples were collected from 23 provinces and autonomous regions in mainland China. Subjects with incomplete responses, and who had died or moved away, were excluded. The dataset of the year 2008 was chosen for the baseline when the SUA collection was initiated. Of the 2305 participants eligible in CLHLS, 1236 completed face-to-face interviews using pre-designed questionnaires and participated in the clinical examinations of SUA levels contributing to cross-sectional analyses. The follow-up study was conducted in 2014, and 420 (34.0%) participants were retained for the longitudinal cohort. Participants at baseline reported frequencies of tea consumption using “almost every day”, “occasionally” or “rarely or never”. Participants were identified as tea consumers if reporting “almost every day” or “occasionally”. Uricase colorimetry was used to measure SUA levels. Both cross-sectional and longitudinal analyses were performed in CLHLS.

### Assessment of covariates

A standardized questionnaire was carried out by trained interviewers on the information regarding demographic variables (i.e., sex and age), education level, life- styles (i.e., smoking, alcohol drinking, and tea consumption), and health condition (i.e., history of diabetes, hypertension, and fasting plasma glucose). Education level was divided into “primary education and below” and “secondary schooling and above”. Smoking status and alcohol drinking status were classified into ‘yes’ and ‘no’. History of diabetes, hypertension, and fasting plasma glucose were classified into “presence” and “absence”. BMI was calculated as weight divided by height squared.

### Statistical analysis

Continuous measurements were expressed as mean ± standard deviation (SD) while categorical variables were presented as percentages. The SUA level differences between tea consumers and non-tea consumers among older adults were compared using the Student’s *t*-test. In the cross-sectional analysis, the relationship between SUA levels and tea-consumption parameters was explored by multivariable linear regression models for three covariate sets (three models respectively). Model 1 is for the basic covariate set adjusted for age and sex; Model 2 is for the extend covariate set adjusted for age, sex, education level, smoking status, and alcohol drinking status; Model 3 is for the full covariate set adjusted for covariates in Model 2 and baseline characteristics of body mass index (BMI), history of diabetes, hypertension and fasting plasma glucose. Regression coefficients (βs) and 95% confidence intervals (CIs) were calculated to assess the strength of the tea-drinking-SUA relationship.

The longitudinal cohort studies were conducted to detect the association between tea-consumption parameters and changes of SUA levels. The linear regression models were adjusted for potential confounders with the similar covariate sets mentioned above. Distributions of SUA levels among sex-subgroups were displayed to examine whether sex acted an important effect. Given the relationship between tea-consumption parameters and SUA levels found in the cross-sectional analysis, a sex-stratified analysis was further conducted to explore the potential gender effect. The multivariable linear regression models were fitted after adjusting for the full covariate set as in Model 3 mentioned above. All statistical analyses were performed using SAS (version 9.4, SAS Institute, Cary, NC, USA). Two-sided *P*-values of less than 0.05 were considered statistically significant.

## Results

In this analysis, participants were from three population-based studies (WGDS, CHNS and CLHLS), and their characteristics were displayed in Table 1. The mean and range of participants’ ages at baseline were 67.7 ± 6.3 (60.0–93.0), 68.9 ± 6.8 (60.0–99.0) and 86.6 ± 12.3 (62.0–110.0) respectively. The category of tea drinking frequencies was by the criterion “6-times-per-week” among the habitual tea drinkers. Types of tea were considered in the WGDS study to explore a potential tea component effect.

**Table 1:** Characteristics of participants aged 60 or above from the three population-based datasets

	WGDS	CHNS	CLHLS
Survey year(s)	2014–2018	2009	2008–2014
Sample size at baseline	4579	2440	1236
Sample size at follow-up	3870	–	420
Mean age at baseline, years (SD)	67.7 (6.3)	68.9 (6.8)	86.6 (12.3)
Age range at baseline, years	60–93	60–99	62–110
Men, n (%)	2200 (48.0)	1149 (47.1)	493 (39.9)
BMI at baseline, mean (SD)	23.3 (2.6)	23.3 (3.7)	20.0 (3.8)
Diabetes at baseline, n (%)	367 (8.0)	331 (13.6)	133 (10.8)
Hypertension at baseline, n (%)	2463 (53.8)	670 (27.5)	173 (14.0)
Habitual tea drinker, n (%)	1571 (34.3)	911 (37.3)	435 (35.2)
Current cigarette smoker, n (%)	1193 (26.1)	621 (25.5)	266 (21.5)
Current alcohol drinker, n (%)	1035 (22.6)	637 (26.1)	233 (18.9)
Fasting plasma glucose at baseline, mmol/L, mean (SD)	5.6 (1.1)	5.7 (1.7)	5.4 (1.9)
Type of tea, n (%)			
Green tea	1408 (89.6)	–	–
Black or Oolong tea	91 (5.8)	–	–
other	72 (4.6)	–	–
Frequency, n (%)			
≥ 6 times/week	1398 (88.99)	717 (78.9)	265 (60.9)
< 6 times/week	173 (11.01)	192 (21.1)	170 (39.1)
Education level, n (%)			
Primary education and below	3954 (86.4)	1749 (71.7)	1165 (94.3)
Secondary schooling and above	625 (13.6)	691 (28.3)	71 (5.7)

*BMI* body mass index; SD, standard deviation, *WGDS* Weitang Geriatric Diseases Study, *CHNS* China Health and Nutrition Survey, *CLHLS* Chinese Longitudinal Healthy Longevity Survey

**Fig. 1:**
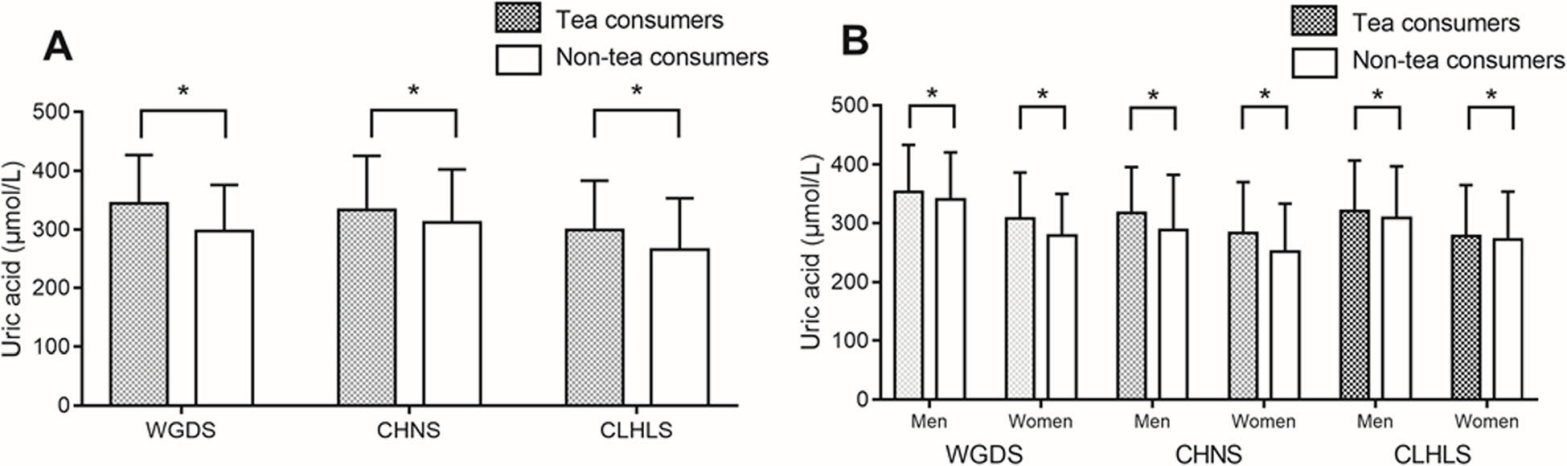
**a** The uric acid levels of tea consumers and non-tea consumers for the WGDS, CHNS and CLHLS datasets. The symbol * indicates significant difference (*P* < 0.05). **b** Comparison between tea consumers and non-tea consumers in the sex-subgroups for the three datasets

The tea consumers had significantly higher SUA levels compared to those without the tea-drinking habit as shown in Fig. 1, and this tendency was consistent in the sex-subgroups as well as the whole datasetsfor all the three studies. Table 2 showed relationships between tea-drinking parameters and SUA levels, and the multiple linear regression analyses were fitted after adjusting for potential confounders. SUA levels were significantly associated with tea drinking status (Yes/No) in all models (*P* ≤ 0.05), agreeing with Fig. 1 above. We found that the participants with high frequencies of tea drinking (no less than 6 times per week) tended to have higher SUA levels compared to those with less tea-drinking frequencies. Significant associations (*P* < 0.05) were found for the adjusted linear regression models in all studies except Model 3 in CHNS. Furthermore, tea type played an important role, as green tea significantly increased SUA levels. For example, the SUA level of green tea increased by 18.69 (95%CI: 12.74, 24.64) compared to other tea types.

**Table 2:** Linear regression coefficients between tea drinking and SUA levels at baseline

	Model 1		Model 2		Model 3	
	*β* (95% CI)	*P* value	*β* (95% CI)	*P* value	*β* (95% CI)	*P* value
WGDS						
Tea drinking status (Yes/No)	18.98 (13.21, 24.75)	< 0.01	14.94 (8.17, 21.70)	< 0.01	17.19 (11.40, 22.97)	< 0.01
Frequency (≥6/< 6)	9.56 (6.55, 12.58)	< 0.01	8.46 (5.40, 11.52)	< 0.01	8.60 (5.51, 11.62)	< 0.01
Tea type (green tea/other)	18.69 (12.74, 24.64)	< 0.01	11.61 (10.58, 22.65)	< 0.01	16.96 (11.01, 22.91)	< 0.01
CHNS						
Tea drinking status (Yes/No)	10.09 (2.74, 17.43)	< 0.01	9.53 (2.18, 16.91)	0.01	7.24 (−0.42, 14.52)	0.05
Frequency (≥6/< 6)	14.37 (0.24, 28.51)	< 0.05	14.00 (−0.15,28.15)	0.05	10.75 (−3.23, 24.72)	0.13
CLHLS						
Tea drinking status (Yes/No)	31.96 (22.16, 41.77)	< 0.01	30.32 (20.50, 40.14)	< 0.01	30.75 (20.96, 40.54)	< 0.01
Frequency (≥6/< 6)	28.05 (16.56, 39.54)	< 0.01	27.09 (15.63, 38.55)	< 0.01	27.95 (16.52, 37.38)	< 0.01

*CI* confidence interval, *WGDS* Weitang Geriatric Diseases Study, *CHNS* China Health and Nutrition Survey, *CLHLS* Chinese Longitudinal Healthy Longevity Survey

Model 1, adjusted for age, sex

Model 2, adjusted for age, sex, education level (primary education and below vs. secondary schooling and above), smoking status (Yes and No), and alcohol drinking status (Yes and No)

Model 3, adjusted for covariates in model 2 and baseline characteristics of body mass index (BMI), history of diabetes (presence vs. absence), hypertension (presence vs. absence), and fasting plasma glucose

**Fig. 2:**
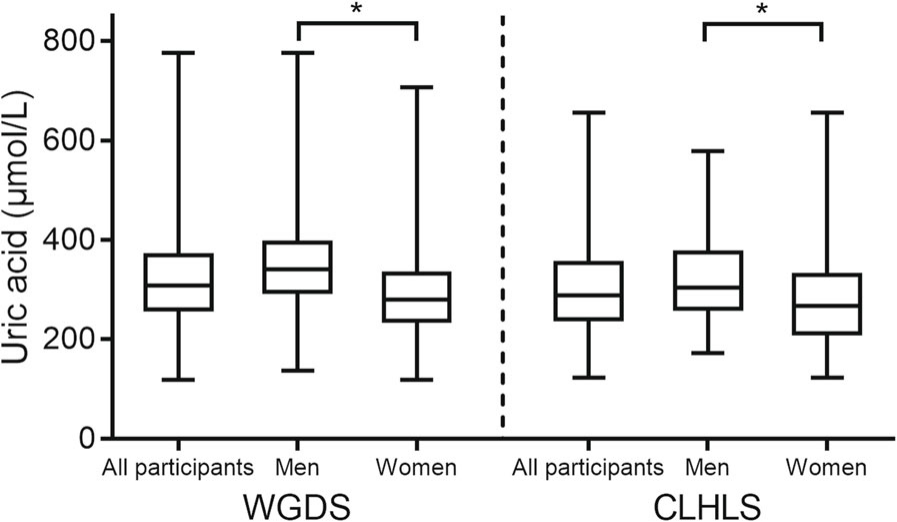
Uric acid boxplots of all participants, men and women at follow-up for the WGDS and CLHLS datasets, respectively

We further examined the relationship between SUA levels and tea-drinking by longitudinal cohort studies on the WGDS and the CLHLS as shown in Table 3. However, the changes of SUA levels had no relationship with tea-drinking parameters, with no statistical significance (*P* > 0.05) for all linear regression models adjusted for covariates such as age, sex, BMI and so on. To further explore the sex effect on longitudinal cohort studies, distributions of SUA levels in the follow-up periods were shown in Fig. 2, where the mean, standard deviation and range were displayed for men, women and the whole datasets. The SUA mean of men was found significantly larger than that of women among the sex-subgroups.

**Table 3:** Linear regression coefficients between tea drinking and SUA levels in the follow-up period

	Model 1		Model 2		Model 3	
	*β* (95% CI)	*P* value	*β* (95% CI)	*P* value	*β* (95% CI)	*P* value
WGDS						
Tea drinking status (Yes/No)	−0.98 (−5.66, 3.69)	0.68	−1.36 (−6.12, 3.40)	0.58	−1.47 (−6.23, 3.29)	0.55
Frequency (≥6/< 6)	−0.46 (−2.90, 1.98)	0.71	−0.66 (−3.14, 1.83)	0.60	− 0.72 (− 3.21, 1.76)	0.57
Tea type (green tea/other)	−2.49 (−7.31, 2.32)	0.31	−2.91 (−7.80, 1.99)	0.25	−3.01 (−7.90, 1.89)	0.23
CLHLS						
Tea drinking status (Yes/No)	−7.51 (−21.01, 5.99)	0.28	−6.78 (−20.44, 6.88)	0.33	−6.27 (− 20.06, 7.51)	0.37
Frequency (≥6/< 6)	−9.67 (− 25.14, 5.80)	0.22	−8.78 (− 20.43, 6.86)	0.27	−8.50 (− 24.26, 7.27)	0.29

*CI* confidence interval, *WGDS* Weitang Geriatric Diseases Study, *CHNS* China Health and Nutrition Survey, *CLHLS* Chinese Longitudinal Healthy Longevity Survey

Model 1, adjusted for age, sex

Model 2, adjusted for age, sex, education level (primary education and below vs. secondary schooling and above), smoking status (Yes and No), and alcohol drinking status (Yes and No)

Model 3, adjusted for covariates in model 2 and baseline characteristics of body mass index (BMI), history of diabetes (presence vs. absence), hypertension (presence vs. absence), and fasting plasma glucose

We further conducted the sex-stratified analysis on cross-sectional studies to explore the sex effect on SUA-tea correlations as shown in Table 4. The linear regression coefficients were from the adjusted models similar to Model 3 at Table 2. The relationships were found significant in gender subgroups in the WGDS and the CLHLS. Compared with Model 3 of Table 2 for the whole datasets, tea-SUA correlations in the sex-stratified analysis maintained significant in the WGDS and the CLHLS (*P* < 0.01), but were weakened in the CHNS for the tea drinking status (*P* > 0.05).

**Table 4:** Linear regression coefficients between tea drinking habits and SUA levels by gender at baseline

	Men		Women	
	*β* (95% CI)	*P* value	*β* (95% CI)	*P* value
WGDS				
Tea drinking status (Yes/No)	12.49 (5.07, 19.91)	< 0.01	20.66 (11.06, 30.27)	< 0.01
Frequency (≥6/< 6)	6.45 (2.68, 10.22)	< 0.01	10.66 (5.29, 16.02)	< 0.01
Tea type (green tea/other)	12.54 (5.22, 19.86)	< 0.01	22.43 (11.49, 33.63)	< 0.01
CHNS				
Tea drinking status (Yes/No)	3.93 (− 2.02, 9.88)	0.20	3.68 (−1.40, 8.76)	0.16
Frequency (≥6/< 6)	3.05 (− 6.78, 12.88)	0.51	−3.23 (−12.90, 6.43)	0.51
CLHLS				
Tea drinking status (Yes/No)	29.01 (13.14, 44.89)	< 0.01	31.93 (19.42, 44.44)	< 0.01
Frequency (≥6/< 6)	26.74 (8.96, 44.51)	< 0.01	28.83 (13.78, 43.87)	< 0.01

*CI* confidence interval, *WGDS* Weitang Geriatric Diseases Study, *CHNS* China Health and Nutrition Survey, *CLHLS* Chinese Longitudinal Healthy Longevity Survey

Adjusted for age, sex, education level (primary education and below vs. secondary schooling and above), smoking status (Yes and No), alcohol drinking status (Yes and No), body mass index (BMI), history of diabetes (presence vs. absence), hypertension (presence vs. absence), and fasting plasma glucose

## Discussion

In the present analyses, we examined relationships between tea consumption and SUA levels for Chinese adults aged 60 years or older. Three large-samplestudies were accessed and compared for cross-sectional analyses, and regions investigated covered from Weitang Town of Suzhou City to the whole mainland of China. Furthermore, two datasets were selected for longitudinal studies with the follow-up periods of 4 and 6 years. In cross-sectional analyses SUA levels were found higher among tea consumers than non-tea consumers at baseline, but no significant associations were observed in longitudinal analyses. Our results added new knowledge to the role of tea-drinking in SUA levels by comparing different statistical analyses on three large-scale samples.

The arguments on associations between tea consumption and SUA levels have been reported in previous studies [15, 16, 26–31]. Some studies demonstrated that SUA levels decreased for tea drinkers [15, 30]. In contrast, the results from two Asia studies (Singapore and Korean) showed positive associations between SUA levels and tea consumption [16, 29]. In addition, no association was reported in other population-based studies from either western or Asian countries [26–28, 31]. The disagreement might be due to different approaches addressing how tea intake influences SUA levels. In rat experiments, green tea was found to decrease SUA levels [15]. Among previous epidemiological studies, cross-sectional analyses found either no association [26, 28] or positive connections [29]. While for longitudinal analyses connections could be positive [16], negative [30], or none [27, 31]. Due to the disagreements from different statistical analyses on various datasets, we conducted both cross-sectional and longitudinal analyses, and compared the studies between three large samples. Our longitudinal analyses showed no correlation between SUA levels and tea intakes, while the correlation appeared in our cross-sectional analyses. This study suggests no statistically significant association between SUA levels and tea intake.

The mechanics of how tea consumption might influence SUA levels have not been determined, and two tea components, tea polyphenols and caffeine, are mostly discussed in experiments [12, 15–17, 26–33]. Tea polyphenols can affect both the production and excretion of SUA [17]. On one hand, catechins and theaflavins are effective inhibitors of XO (xanthine oxidase) to the production of UA, which leads to SUA decrease [15]. On the other hand, catechins inhibit expression of OATP1 (organic anion transporter) [32], a secreting protein for UA excretion, and they could cause an upward trend of SUA [34]. Another component caffeine (1,3,7-trimethyl-xanthine) is a methyl-xanthine, which is the major component of coffee as well. There has been no clear explanation on how caffeine affects SUA. Caffeine is metabolized by demethylation into the urinary metabolite l-methylxanthine, which was found inhibitive effect on XO in in-vitro and vivo experiments of rats [35]. In other rat experiments caffeine was found inducing renal deterioration and high insulin sensitivity, which resulted in increase of SUA levels [36]. This could be related to the positive association between SUA levels and caffeine intake found in females in the epidemiologic study [29]. Furthermore, during the follow-up period of 4–6 years, possible changes of health conditions could influence SUA levels. Hyperuricemia was found associated with cardiovascular disease, renal disease, metabolic diseases, diabetes, hypertension and other diseases [37].

The strength of this study includes its prospective design and the population-based large sample size. It also has several limitations. First, the study samples consisted primarily consisted of older Chinese adults. The homogeneity of the study participants minimized confounding by socioeconomic status, but the generalizability of our data to other populations segments may be limited, such as younger generations and other racial or ethnic groups. Second, the information on tea consumption was self-reported and collected by questionnaires, which may lead to misclassifications of exposure. Because of the prospective nature of the analysis, these misclassifications tended to have attenuated our results towards the null. Third, although we controlled for a wide range of covariates including lifestyle factors, the possibility of unmeasured and residual confounding cannot be fully excluded. We did not assess whether participants had renal dysfunction, obesity, medications, thyroid, and related disorders, which may has changed the levels of SUA.

## Conclusions

In conclusion, results from these three large and well-established population-based studies demonstrated a significant connection between tea consumption and SUA levels at baseline, but these provided no support for the longitudinal association in the follow-up of 4–6 years. Misclassification bias could have influenced the longitudinal association and the direction of the bias would be more likely towards the null in our study. Additional well-designed cohort studies with more precise measurement of tea consumption (e.g. cup capacity and tea concentration) and longer follow-up durations are needed to confirm our findings.

## Data Availability

The datasets used and/or analyzed during the current study are available from the corresponding author on reasonable request.

## Abbreviations

SUA: serum uric acid
WGDS: Weitang Geriatric Diseases Study
CHNS: China Health and Nutrition Survey
CLHLS: Chinese Longitudinal Healthy Longevity Survey
